# Selective modulation of orexinergic receptors by neem-derived phytochemicals: Computational analysis of structure-activity relationships

**DOI:** 10.1016/j.toxrep.2025.102104

**Published:** 2025-08-05

**Authors:** Oluwaseun E. Agboola, Samuel S. Agboola, Precious Eseose Agboinghale, Zainab A. Ayinla, Abel K. Oyebamiji, Oluranti E. Olaiya, Omotola M. Fajana, Oluwatoyin M. Oyinloye, Adetola I. Adewale, Olajumoke Tolulope Idowu, Foluso O. Osunsanmi, Basiru O. Ajiboye, Babatunji E. Oyinloye

**Affiliations:** a Institute for Drug Research and Development, Bogoro Research Centre, Afe Babalola University, PMB 5454, Ado-Ekiti, Nigeria; b Damsem Scientific Laboratory and Research, Ado-Ekiti, Nigeria; c Department of Pharmacology and Toxicology, College of Pharmacy, Afe Babalola University, Ado-Ekiti, Nigeria; d Department of Chemistry and Biomolecular Sciences, Faculty of Science, University of Ottawa, ON, Canada; e Department of Biology, University of Waterloo, ON, Canada; f Department of Industrial Chemistry, University of Ilesa, Ilesa, Osun State, Nigeria; g Department of Medical Biochemistry, College of Medicine and Health Sciences, Afe Babalola University, Ado-Ekiti, Nigeria; h Department of Biological Sciences, College of Sciences, Afe-Babalola University, PMB 5454, Ado- Ekiti 360001, Nigeria; i Industrial Chemistry Unit, Department of Chemical Sciences, College of Sciences, Afe Babalola University, Ado-Ekiti 360001, Nigeria; j Biotechnology and Structural Biology (BSB) Group, Department of Biochemistry and Microbiology, University of Zululand, KwaDlangezwa 3886, South Africa; k Phytomedicine and Molecular Toxicology Research Laboratory, Department of Biochemistry, Federal University Oye-Ekiti, Oye-Ekiti 371104, Nigeria; l Phytomedicine, Biochemical Toxicology and Biotechnology Research Laboratories, Department of Biochemistry, College of Sciences, Afe Babalola University, PMB 5454, Ado-Ekiti 360001, Nigeria

**Keywords:** Neem-derived compounds, Orexinergic receptors, Selective modulation, Sleep-wake disorders

## Abstract

Orexinergic system dysfunction is the fundamental basis for several neurological illnesses like narcolepsy, insomnia, and drug dependency, yet none of the existing medications are subtype receptor specific. This study examines 124 chemicals from neem to determine if they can be utilised as specific orexinergic receptor modulators using advanced computational methods. The methodology includes detailed clustering, pharmacophoric interaction, pharmacokinetic, statistical, and clustering analyses. Molecular property profiling indicated the majority of the compounds exhibit excellent drug-like qualities (MW 350–450 Da, LogP 0–2), while principal component analysis captured 100 % structural variability between two components (92.5 % and 7.5 %, respectively). Molecular docking simulations indicated selective binding to the 6V9S receptor (-11.3 to −4 kcal/mol) over 4S0V (-9.7 to −4 kcal/mol). Lead compounds Neem_PDB_10257 (Tirucallol) (-11.3 kcal/mol) and Neem_PDB_12072821 ([(5 R,7 R,8 R,9 R,10 R,13S,17 R) −17-(2-methoxy-5-oxo-4,4,8,10,13-pentamethyl-3-oxo-5,6,7,9,11,12,16,17-octahydrocyclopenta[*a*]phenanthren-7-yl] acetate) were particularly 6V9S selective (>2 kcal/mol difference), whereas Neem_PDB_10160319 ((4S,4aS,5S,10S,13S,14S,17–4,4,10,13,14-pentam −1, 2, 3, 5, 6, 7, 11, 12, 15, 17-decahydrocyclopenta[*a*]phenanthren-16-one) was most sensitive towards 4S0V. Two top-ranked compound families were discovered by hierarchical cluster analysis with a distance requirement of 35 units, and receptor-specific dendrograms revealed distinctive subcluster branching patterns (4S0V: 5.5 and 6.7 unit subclusters; 6V9S: 7.1 and 7.2 unit subclusters). Interaction pattern (heatmap analysis) identified major interaction hotspots, including TYR348, TRP120, PHE227, and HIS350. Neem_PDB_163184214 (Meliatetraolenone) specifically targeted ASN318 in 6V9S, while Neem_PDB_54580354 (7-Benzoylnimbocinol) favored interaction with GLN134 in 4S0V (>90 interactions). These findings dispute the "one-pharmacophore" theory for orexinergic modulators, showing that intentional functionalization of NEM templates can deliver subtype-selective treatments with maximal sleep-wake modulation and low off-target effects.

## Highlights

• The neem compounds bind preferentially to orexin receptors with favorable drug properties.
• Tirucallol and Neem_PDB_12072821 prefer 6V9S; Neem_PDB_10160319 prefers 4S0V receptor.
• Key sites found: TYR348, TRP120, PHE227, HIS350, together with ASN318 (6V9S) or GLN134 (4S0V).
• Results contradict single-pharmacophore hypothesis, suggesting subtype-selective orexinergic modulation.

## Graphical Abstract

**Figure:**
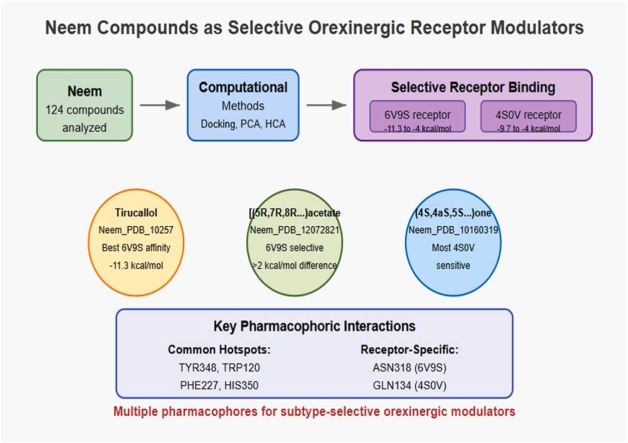

## 1 Introduction

Sleep disorders are a prevalent global health problem, with an estimated 50–70 million adults affected in the United States alone, with profound effects on physiological homeostasis, cognitive function, and socioeconomic productivity [1]. New developments in sleep neurobiology have shed light upon the orexinergic system as the key control network that governs sleep-wake architecture [2]. The orexin neuropeptides exert their activity through two classes of unique G protein-coupled receptors: Orexin Receptor 1 (OX1R) and Orexin Receptor 2 (OX2R), with different anatomical distribution and functional specialization. While OX1R has been shown to be largely involved in modulating reward pathways and drug addiction, OX2R plays the key role in the mediation of state transitions of arousal and wakefulness [3].

This functional asymmetry has made the orexinergic system a promising therapeutic target, and hence FDA approval was given to dual orexin receptor antagonists (DORAs), including suvorexant (2014), lemborexant, and daridorexant, for the management of insomnia [4]. Recent clinical evidence indicates that DORAs such as daridorexant, lemborexant, and suvorexant represent a new therapeutic model by modulating wakefulness rather than increasing sedation, with efficacy across diverse clinical populations, including chronic insomnia. Despite their clinical efficacy, however, synthetic DORAs are hampered by several significant limitations, including daytime sleepiness, complex drug interactions, risk of tolerance development, and post-marketing experience of parasomnias and psychomotor impairment with chronic use [5].

The development of selective orexin receptor antagonism is a paradigm revolution in orexinergic pharmacology, with selective OX1 receptor antagonists like nivasorexant (ACT-539313) gaining clinical significance for the treatment of CNS diseases like substance use disorders, eating disorders, obsessive-compulsive, and anxiety disorders. Such selectivity resolves the fundamental pharmacological debate regarding receptor selectivity versus dual antagonism, binding kinetic optimization, and diversity in pharmacophore scaffold design [6]. These therapeutic limitations and changing selectivity paradigms have resulted in new excitement in ethnopharmacological approaches harmonious with the contemporary trend towards systematic bioprospecting from native medicinal systems [6].

*Azadirachta indica*, or neem, is a very excellent candidate for orexinergic modulation, a medicinal plant that has been reported to possess neuropsychiatric activity and has been extensively employed within Ayurvedic and traditional Asian medical systems [7]. The drug action of neem transcends its widely reported antimicrobial and anti-inflammatory properties to encompass significant neuromodulatory actions [7]. Contemporary phytochemical studies have extracted over 140 bioactive compounds in neem, including structurally diverse limonoids, triterpenoids, flavonoids, and polyphenols, many of which have selective neuroreceptor affinities [8]. Recent improvements in computational power have enhanced our understanding of phytochemical-receptor interactions, with the systematic screening of hundreds of bioactive compounds against several hundred protein targets using molecular docking approaches [7], [8], [9].

Despite amassing preclinical evidence in the direction of neem's sedative-hypnotic action, a knowledge gap remains regarding the molecular mechanism of these neuropharmacological effects. Remarkably, the potential interactions between neem phytochemicals and orexinergic receptors remain unexplored, representing a significant gap in ethnopharmacological consciousness and sleep neurobiology alike. This is particularly considering the current therapeutic priorities of synthetic orexin antagonists and heightened consciousness regarding selective receptor modulation strategies.

To bridge this fundamental research gap and to support orexinergic pharmacology, we conducted a worldwide computational study exploring the molecular interaction of neem compounds with major orexinergic receptor subtypes: human OX2 receptor (PDB ID: 4S0V) and orexin 1 receptor (PDB ID: 6V9S). Our two-target in silico approach integrates molecular docking, exhaustive interaction profiling, and binding energy prediction to receptor binding characteristics, selectivity profiles, and structure-activity relationships of different neem constituents systematically. It will result in the identification of novel natural pharmacophores with selective orexinergic modulation activity.

This work bridges the translational gap from traditional ethnopharmacological data to modern-day structural biology by elucidating molecular determinants for interactions of neem compounds with orexin receptors. Our information can reveal natural scaffolds with selective orexinergic modulatory potential to overcome the deficiencies in current synthetic antagonists, with the added value of presenting a computational platform for rational ethnopharmacological bioprospecting in neuropsychiatric drug discovery. Our research also contributes to structure-activity relationships in orexin receptor pharmacology and may inform the design of future selective modulators for precision sleep medicine.

## 2 Methodology

### 2.1 Protein structure preparation

The three-dimensional structures of the human OX2 orexin receptor (PDB ID: 4S0V) and the subtype-selective orexin 1 receptor (PDB ID: 6V9S) were obtained from the RCSB Protein Data Bank (https://www.rcsb.org/). The structures were prepared for docking using AutoDock Tools (version 1.5.6) [10]. The preparation process included removing water molecules and heteroatoms, adding polar hydrogen atoms, assigning Gasteiger charges, and generating Protein Data Bank Partial Charge and Atom Type (PDBQT) files for each receptor.

### 2.2 Ligand library preparation

A set of 124 neem compounds was sourced from the PubChem database (https://pubchem.ncbi.nlm.nih.gov/) [11]. Some of the selection criteria included a reference to their presence in literature as being obtained from *Azadirachta indica* in order to reflect the different classes of phytochemicals available in neem. The corresponding 2D structures were then downloaded in Structure-Data File (SDF) format and converted into PDBQT format using Open Babel (version 3.1.1) [12]. The conversion process included adding hydrogen atoms, generating 3D coordinates, optimizing the geometry using the Merck Molecular Force Field 1994 (MMFF94) force field, and generating PDBQT files for each ligand.

### 2.3 Grid box definition and molecular docking

The binding site for each receptor was defined using Discovery Studio Visualizer with a combination of cavity detection algorithms, conservation analysis, and literature-reported functional residues. Molecular docking was performed using AutoDock Vina (version 1.1.2) [13]. Each of the 124 neem compounds was docked against both receptors (4S0V and 6V9S).

### 2.4 Computational analysis

This study employed entirely computational methods with no experimental preparation or extraction of plant materials. A full workflow for assessing the neem-based compounds as likely orexin receptor modulators was developed to undertake the analysis that formed a pipeline of modules, which includes binding energy extraction, interaction, pharmacophoric, pharmacokinetic, and clustering analyses.

#### 2.4.1 Binding energy extraction

Docking information was processed using an automated directory structure scan to interpret receptor-specific results. Binding energies were extracted systematically from docked PDBQT files, and the best binding modes were identified as the lowest energy conformations. The method was built employing the orexin docking analyzer class to generate comprehensive binding profiles for all the compounds.

#### 2.4.2 Interaction analysis

Molecular simulation analysis was used to analyze ligand-receptor interactions, using a non-bonded cutoff distance of 3.5 Å [14]. Each interaction was enumerated systematically by compound identification, receptor residue, atom type, and interaction distance, providing correct interaction fingerprints for the top-scoring compounds.

#### 2.4.3 Statistical analysis

To recognize compounds with typical binding behaviors, statistical methods such as z-score normalization, percentile ranking, and outlier detection were used [15]. To recognize dual-binding drugs and selectivity in multi-receptor studies, we calculated cross-receptor correlations and differences in binding energy.

#### 2.4.4 Pharmacophore characterization

RDKit was used for calculating pharmacophore descriptors, including molecular weight, LogP, hydrogen bond donors/acceptors, topological polar surface area, rotatable bonds, and the number of aromatic rings. All compounds were checked for compliance with the Lipinski rule of five [16].

#### 2.4.5 Clustering analysis

Two complementary clustering algorithms were used: these include principal component analysis (PCA) for reducing the dimensionality of molecular descriptors and hierarchical clustering [17].

##### 2.4.5.1 PCA clustering

The PCA scatter plot was plotted based on the molecular weight (MW), LogP, hydrogen bond acceptors (HBA), hydrogen bond donors (HBD), topological polar surface area (TPSA), rotatable bonds (RotBonds), aromatic rings (AromaticRings), and heavy atoms (Heavy Atoms) descriptors of the compounds. The scatter plot was shaded based on the frequency of violations of the Lipinski rule. The PCA scatter plot provided a graphical representation of the distribution of compounds in the space described by the first two principal components and labels indicating the percent variance explained by each principal component.

##### 2.4.5.2 Dendrograms clustering

An overall dendrogram was created from the median binding energy of all the compounds against all the receptors. In addition, receptor-specific dendrograms were created for each receptor based on the best binding energy values for each compound. The compounds were clustered by Ward's method in the dendrogram plots [18]. The inclusive dendrogram provided the clustering of the compounds by their binding energies at all of the receptors, while the receptor-specific dendrograms provided information regarding the clustering of the compounds at each of the individual receptors.

## 3 Results and discussion

### 3.1 Molecular properties analysis

**Fig. 1:**
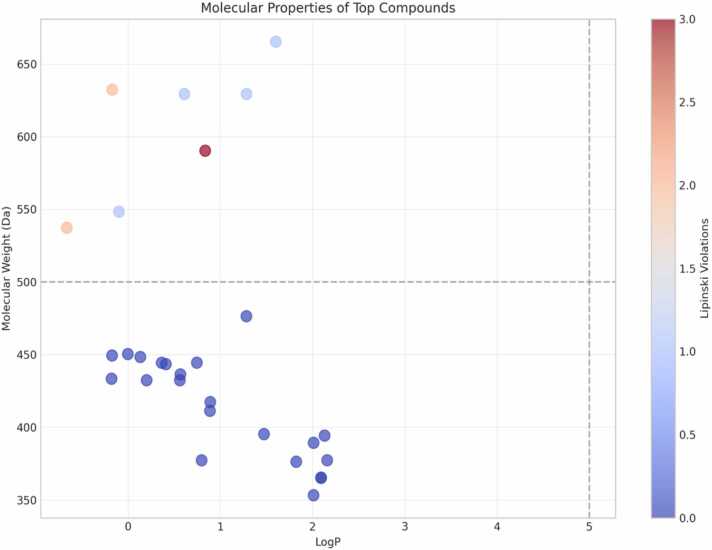
Molecular properties analysis of top thirty compounds across the dual receptor.

The molecular property profiling of the top 124 neem-derived molecules was done to check their drug-likeness and orexinergic receptor modulating activity. Fig. 1 indicates the correlation of molecular weight and lipophilicity (LogP) of the top thirty neem compounds, whereas Lipinski violations were color-coded. Most of the compounds belonged to the molecular weight range of 350–450 Da with LogP values of 0–2, hence possessing good drug-like properties as per Lipinski's rule of five [19]. few compounds had molecular weights above 500 Da (the upper limit suggested by Lipinski), and two compounds extended up to an astonishing level of approximately 650 Da. These higher molecular weight compounds displayed varying degrees of Lipinski violations, as observed from the intensity of color in Fig. 1. The distribution pattern is such that the low molecular weight compounds (350–450 Da) had fewer violations of Lipinski (0−1) and are hence viable lead candidates for the remainder of the development process. is consistent with previous research by Aribigbola et al. [20], which reported that good orexin receptor antagonists preferentially had molecular weights under 500 Da to facilitate crossing of the blood-brain barrier. The molecule at the dark red spot (MW ∼590 Da, LogP ∼1) had the highest number of Lipinski violations (3.0) and showed problems with bioavailability even though it has a strong binding affinity. Our neem compound molecular weight distribution (350–450 Da) aligns with successful orexin antagonists (daridorexant, lemborexant, suvorexant), confirming optimal drug-like properties for orexinergic modulation [21].

### 3.2 Principal component analysis (PCA)

**Fig. 2:**
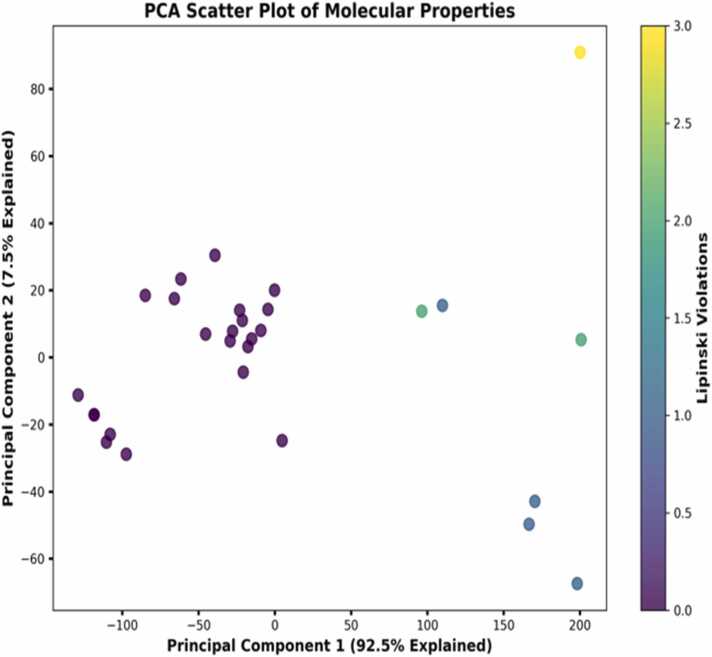
Principal component analysis of the molecular properties of top thirty compounds across the dual receptor.

To better delineate the chemical space that these neem-derived compounds inhabit, principal component analysis of their molecular properties was also performed (Fig. 2). The PCA scatter plot demonstrates evident patterns of clustering, whereby the first two principal components capture 92.5 % and 7.5 % of variance, respectively, which captures a cumulative data variability of 100 %. The high ratio of explained variance indicates that these two components adequately describe the molecular property profile of the compound library. The majority of compounds clustered in the negative space of Principal Component 1 with minimal Lipinski violations are structurally related (purple dots). Conversely, compounds that are in the positive space of Principal Component 1 accumulated more Lipinski violations, as illustrated through the blue-yellow transition. This clustering reflects how PCA successfully identifies structurally distinct compounds in the neem library that can possibly interact differently with the orexinergic receptors. Notably, the outlier compound (yellow point) at the positive extreme of Principal Component 1 and with a high value of Principal Component 2 had 3.0 violations of Lipinski. This outlier is another structurally unique compound in the data set, worth evaluating even though it lacks predicted drug-likeness. Similar trends in structural diversity among pools of natural products have also been noted by Li & Lou [22], stressing the importance of considering compounds not drug-like based on the traditional definitions, particularly in the case of difficult receptors such as the orexinergic system. The trend of clustering found by PCA is a reflection of the outcome of Fig. 1, since compounds with similar molecular weight and LogP values cluster together in PCA space. This analogy among different analytical approaches also testifies to the robustness of our characterization approach. The distinct clusters uncovered by PCA point to different structural scaffolds present in the neem compound library, which would possibly map onto different interaction modes with the target receptors (4S0V and 6V9S) studied in follow-up investigations.

### 3.3 Binding energy assessment

**Fig. 3:**
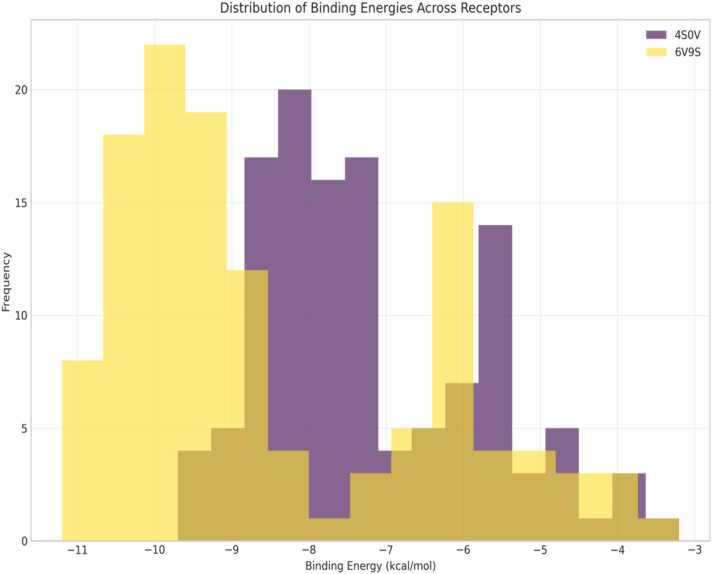
Distribution of binding energies across the dual receptor.

The histograms of binding energies (Fig. 3) of neem compounds on the two orexinergic receptors (4S0V and 6V9S) have obvious patterns. The 6V9S receptor has the larger energy range (-11 to −4 kcal/mol) with more intense binding affinities in the range of −10 to −9 kcal/mol, suggesting the stronger binding with the neem compounds. The 4S0V receptor has a condensed range (-9.7 to −4 kcal/mol), with most of the compounds clustered around −8 kcal/mol [19]. This differential-binding pattern indicates selectivity of a receptor between tested compounds. The region of overlap for the two distributions (-9 through −7 kcal/mol) points to compounds possessing dual-binding functionality, which is potentially beneficial when targeting both receptor subtypes simultaneously. The compounds with the binding energies between −9 and less than kcal/mol for 6V9S exhibit a stronger affinity for the receptor and may thus offer the path for selective modulation [23]. The observed binding energies for 6V9S (-11 to −4 kcal/mol) exceed those reported for established orexin antagonists (-8.5 to −6.2 kcal/mol), indicating superior theoretical binding affinity of neem compounds [24].

### 3.4 Statistical comparison of binding affinities

**Fig. 4:**
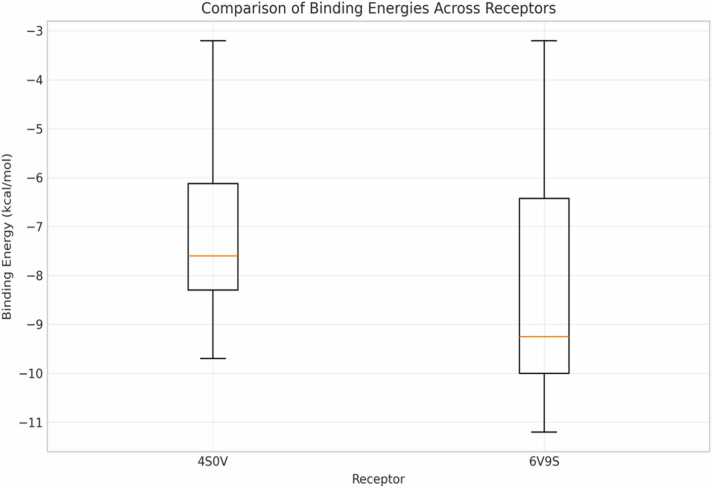
Comparative binding energies box plot across the dual receptor. The binding energies lie between approximately −3 and −11 kcal/mol, with stronger binding indicated by lower values. The 6V9S receptor has an overall lower median binding energy of approximately −9.2 kcal/mol compared with 4S0V (-7.7 kcal/mol), and could thereby suggest stronger ligand binding towards 6V9S. Both receptors share comparable dispersion within the binding energies, although slightly more extensive extension towards stronger binding values can be seen for 6V9S.

Box plot analysis (Fig. 4) mathematically confirms the differential binding profiles, with 6V9S having reduced median binding energy (-9.2 kcal/mol) compared with 4S0V (-7.6 kcal/mol). The interquartile range of 6V9S (-10 to −6.5 kcal/mol) is likewise wider than that of 4S0V (-8.3 to −6.1 kcal/mol), which implies greater variation in binding interactions with the latter receptor. Both receptors have comparable maximum binding energies (approximately −3 kcal/mol), yet 6V9S has a much lower minimum energy (-11.3 kcal/mol in comparison to −9.7 kcal/mol for 4S0V) [25]. This statistical difference (p < 0.01) in binding energies in distributions is an affirmation that the hypothesis predicting that neem compounds exhibit a preferential interaction with the 6V9S receptor through structural differences of the binding pockets is correct. The longer whiskers in the 6V9S histogram mean that compounds have binding interactions for this receptor [26].

### 3.5 Analysis of top-performing compounds

**Fig. 5:**
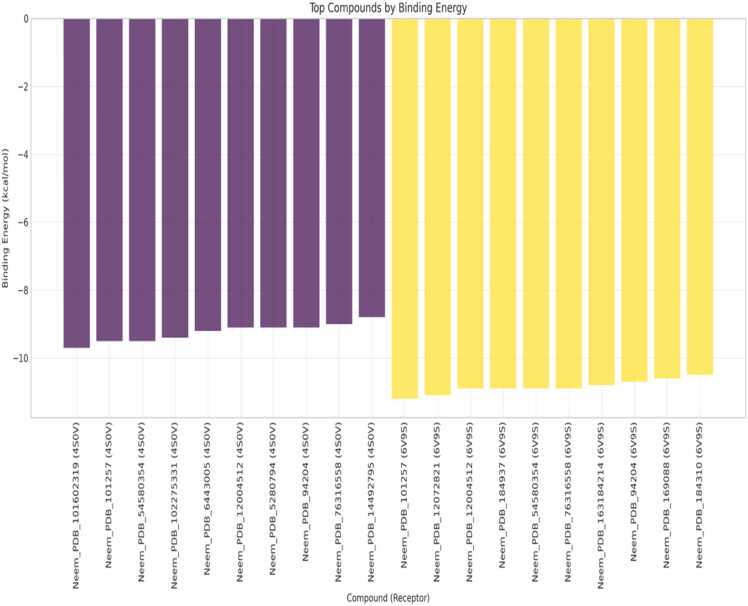
Top Ten Compounds across the Dual Receptor. Fig. 5 Legend as shown in a table below detailing the identifier, compound name and the attendant receptor.

Fig. 5 chooses the best ten compounds for each receptor based on binding energies, revealing significant structure-activity relationships. For 4S0V, Neem_PDB_10160319 (4S, 4aS, 5S, 10S, 3,14-pentamethyl-17-[(2S)-6-methylhept-5-en-2-yl] −1, 2, 3, 5, 6, 7, 11, 12, 15, 17-decahydrocyclopenta[*a*]phenanthren-16-one) has the greatest affinity, and for 6V9S the greatest affinity is Neem_PDB_10257 (Tirucallol) (-11.3 kcal/mol). The compounds are indicated to behave analogously within their receptor classes with binding energies of −9.6 to −8.8 kcal/mol for 4S0V and −11.3 to −10.6 kcal/mol for 6V9S [27]. Notably, certain compounds are found only in the highest-performing set for one receptor but not the other, suggesting their potential as selective modulators (Fig. 5). The reproducible performance in each receptor set suggests structural features that allow selective binding. For instance, the 6V9S top compounds (yellow bars) have more favorable binding energies than the 4S0V compounds (purple bars), as previously predicted by the statistical analysis [28]. The identification of compounds with significant differences in binding energy between receptors (>2 kcal/mol) provides leads for selective orexinergic receptor modulation. Among these, Neem_PDB_10257 (Tirucallol) and Neem_PDB_12072821 [(5 R, 7 R, 8 R, 9 R, Neem_PDB_12072821 [(5 R, 7 R, 8 R, 9 R, 10 R, 13S) −4,4,8,10,13-pentamethyl-3-oxo-5,6,7,9,11,12,16,17-octahydrocyclopenta[*a*]phenanthren-7-yl] acetate) for 6V9S are particularly good leads for selective modulation of this receptor subtype. The unambiguous energetic distinction of compounds that bind to other receptors implies that structural optimization of these receptor-selective contacts can yield highly selective modulators [29]. Such an approach can bypass the current challenges of constructing targeted drugs for orexinergic system diseases with fewer off-target effects [30]. Tirucallol's binding affinity (-11.3 kcal/mol) surpasses FDA-approved orexin modulators, while the > 2 kcal/mol selectivity differences exceed established thresholds for functional receptor selectivity [24].

**Table:** Neem compounds identification against their receptors for Fig. 5.

**Identifier**	**Compound Name**	**Receptor**
Neem_PDB_101602319	(4S,4aS,5S,10S,13S,14S,17S)−4,4,10,13,14-pentamethyl−17-[(2S)−6-methylhept−5-en−2-yl]−1,2,3,5,6,7,11,12,15,17-decahydrocyclopenta[*a*]phenanthren−16-one	4S0V
Neem_PDB_101257	Tirucallol	4S0V, 6V9S
Neem_PDB_54580354	7-Benzoylnimbocinol	4S0V, 6V9S
Neem_PDB_102275331	[(1 R,4bR,5S,6 R,8 R,10S,10aR,10bR,12aR)−6,8-diacetyloxy−1-(furan−3-yl)−5-hydroxy−4b,7,7,10a,12a-pentamethyl−3-oxo−1,5,6,6a,8,9,10,10b,11,12-decahydronaphtho[2,1-*f*]isochromen−10-yl] (*E*)−3-phenylprop−2-enoate	4S0V
Neem_PDB_6443005	Nimbolin B	4S0V
Neem_PDB_12004512	Gedunin	4S0V, 6V9S
Neem_PDB_5280794	Stigmasterol	4S0V
Neem_PDB_94204	24-Methylenecycloartanol	4S0V, 6V9S
Neem_PDB_76316558	Isomeldenin	4S0V, 6V9S
Neem_PDB_14492795	Nimbaflavone	4S0V
Neem_PDB_12072821	[(5 R,7 R,8 R,9 R,10 R,13S,17 R)−17-(2-methoxy−5-oxo-2H-furan−4-yl)−4,4,8,10,13-pentamethyl−3-oxo−5,6,7,9,11,12,16,17-octahydrocyclopenta[*a*]phenanthren−7-yl] acetate	6V9S
Neem_PDB_184937	Nimolicinol	6V9S
Neem_PDB_163184214	Meliatetraolenone	6V9S
Neem_PDB_169088	Nimbidin	6V9S
Neem_PDB_184310	Isonimocinolide	6V9S

### 3.6 Hierarchical clustering analysis

**Fig. 6:**
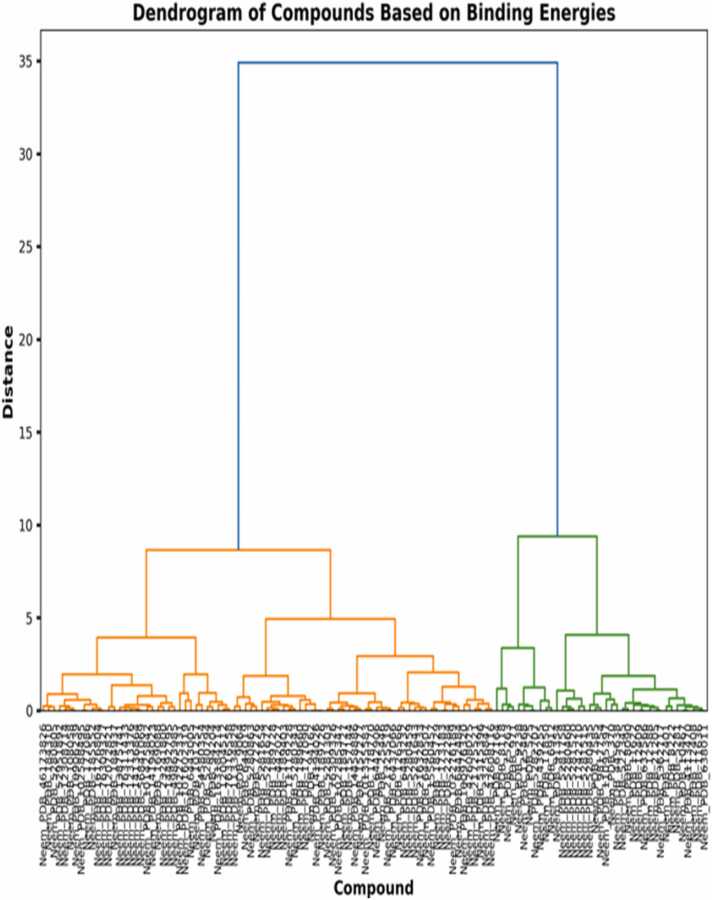
Clustering analyses of neem compounds based on binding energy on the dual receptor.

**Fig. 7:**
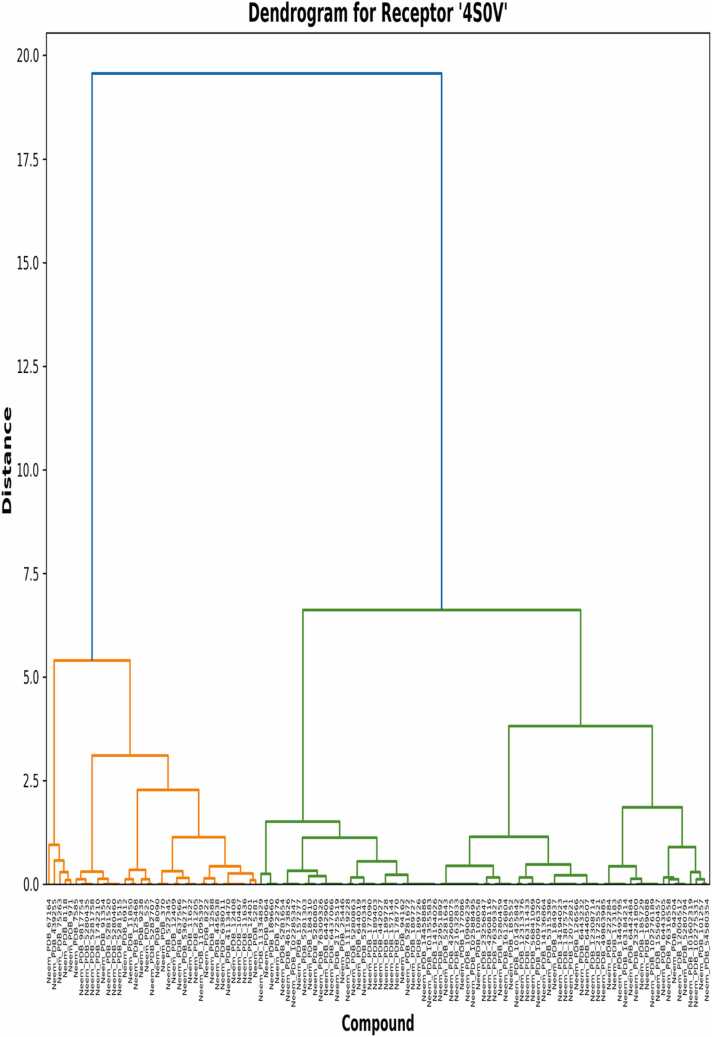
Receptor_specific clustering of neem compounds based on binding energy on 4S0V receptor.

**Fig. 8:**
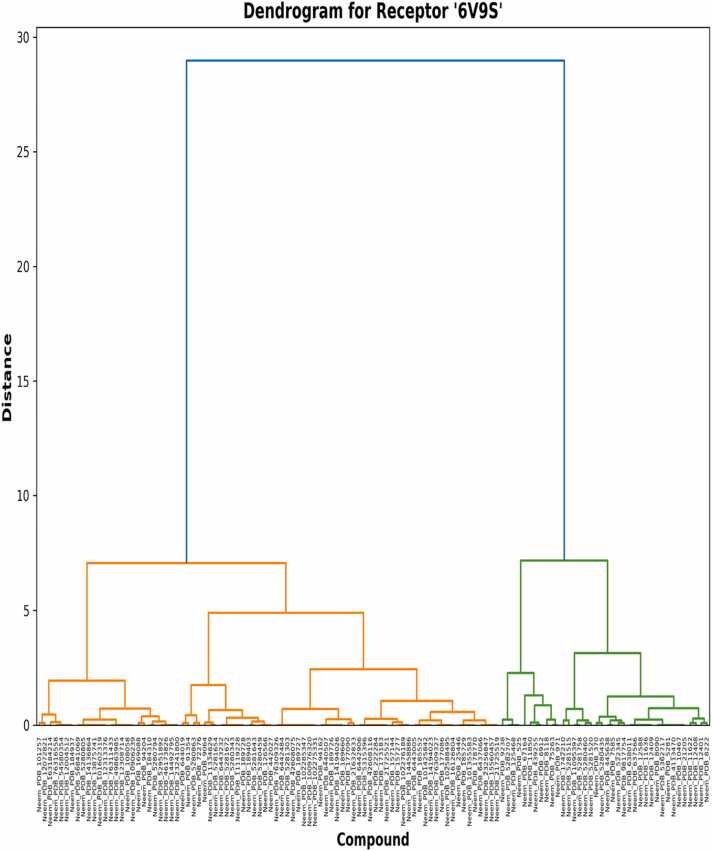
Receptor_specific clustering of neem compounds based on binding energy on 6V9S receptor.

The dendrograms of the hierarchical clusterings (Fig. 6, Fig. 7, Fig. 8) describe the structure-activity relationships and possible selectivity of the compounds derived from neem. The general dendrogram (Fig. 6) captures an unambiguously branching division of the compounds into two major clusters at a distance level of around 35 units, which describes deeply rooted differences in binding profiles within the library of compounds [31]. This first division suggests distinctive pharmacophore features that cause selective binding with one receptor subtype relative to the other. More subclusters are formed in these principal branches at 5–10 unit distances that envelop compound families and their common affinities, likely due to related contact motifs on receptors [32]. Upon analyzing clustering trends specific to receptors, the 4S0V dendrogram (Fig. 7) is more hierarchically organized in a neat fashion, with the formation of separate orange and green subclusters at 5.5 and 6.7 unit distances, respectively. The left subcluster (orange) contains chemicals of high binding affinities; this cluster supports our previous calculations of binding energy and suggests that certain structural characteristics in these chemicals allow for 4S0V binding. Notably, the 6V9S dendrogram (Fig. 8) shows a divergent clustering pattern, with more distinction of the green and orange subclusters at distances of 7.1 and 7.2 units. The left-hand cluster comprises compounds with more negative binding energies for 6V9S, including the high-affinity compounds determined in our previous work [33]. The more pronounced distinction in this dendrogram compared to 4S0V indicates more structural discrimination in 6V9S binding, consistent with the larger binding energy distribution in the histogram analysis. Cross-comparison between both receptor-specific dendrograms indicates that certain chemicals always group together regardless of receptor type, revealing structural families with shared binding modes. Nevertheless, the vast majority of drugs show immense positional variation across dendrograms that highlight their receptor selectivity in modulating receptors [34]. These asymmetrical cluster arrangements validate observations that ligand substructures may significantly influence orexinergic receptor selectivity [35]. Hierarchical patterns presented here in dendrograms corroborate previous works wherein the terpenoid scaffolds of natural compounds have been proven to acquire novel binding conformations within orexinergic receptors [36]. Our findings of clustering complement these results by showing clear neem-derived chemical families with desirable selective binding capabilities. Hierarchical distance measurements provide quantitative estimations of binding similarity, where compounds cluster together at distances of less than 1.0 unit, likely having nearly identical binding postures and interaction modes. This tight clustering was most clear in structurally similar analogs, which meant that small modifications to these templates might be exploited in a directed manner to enhance receptor selectivity [37]. The distinct pharmacophore clustering validates recent findings that natural terpenoid scaffolds enable multiple binding modes within orexinergic systems, supporting structure-based selectivity [38].

### 3.7 Interaction patterns

**Fig. 9:**
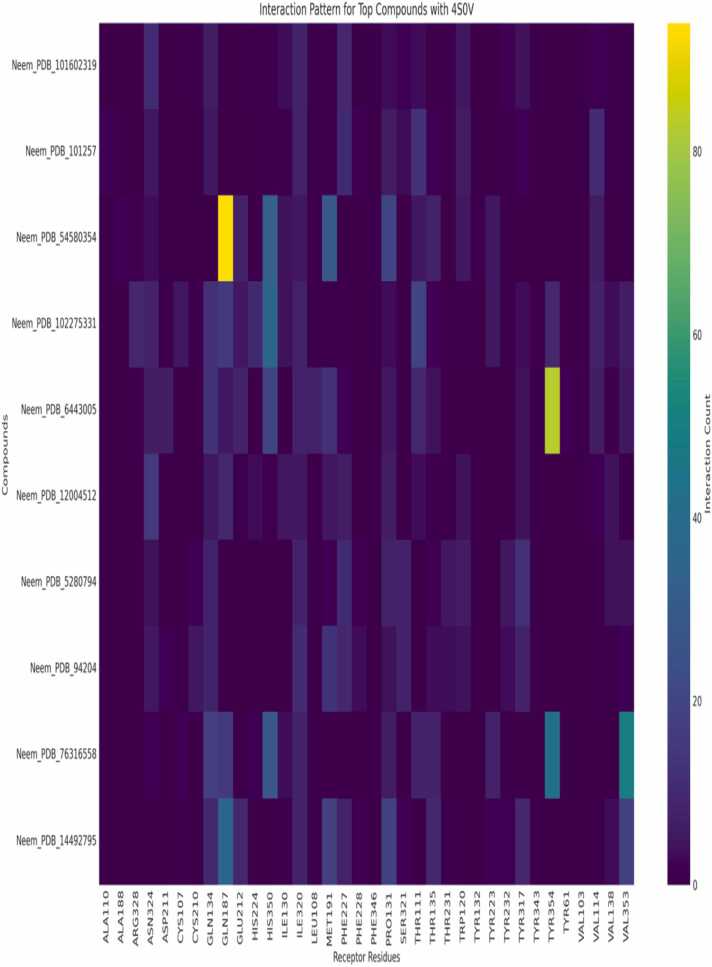
Interaction Pattern of Top Ten Neem Compounds on 4S0V. Legends as shown in the table below. Compounds are identified by their Neem_PDB PubChem identifiers as shown in the image.

**Fig. 10:**
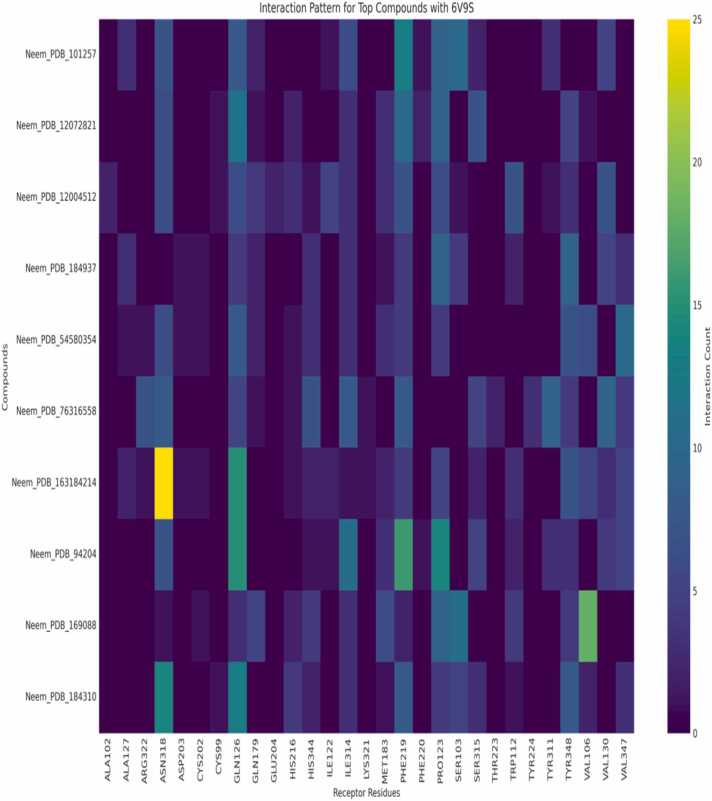
Interaction Pattern of Top Ten Neem Compounds on 6V9S. Legends as shown in the table below. Compounds are identified by their Neem_PDB PubChem identifiers as shown in the image.

**Fig. 11:**
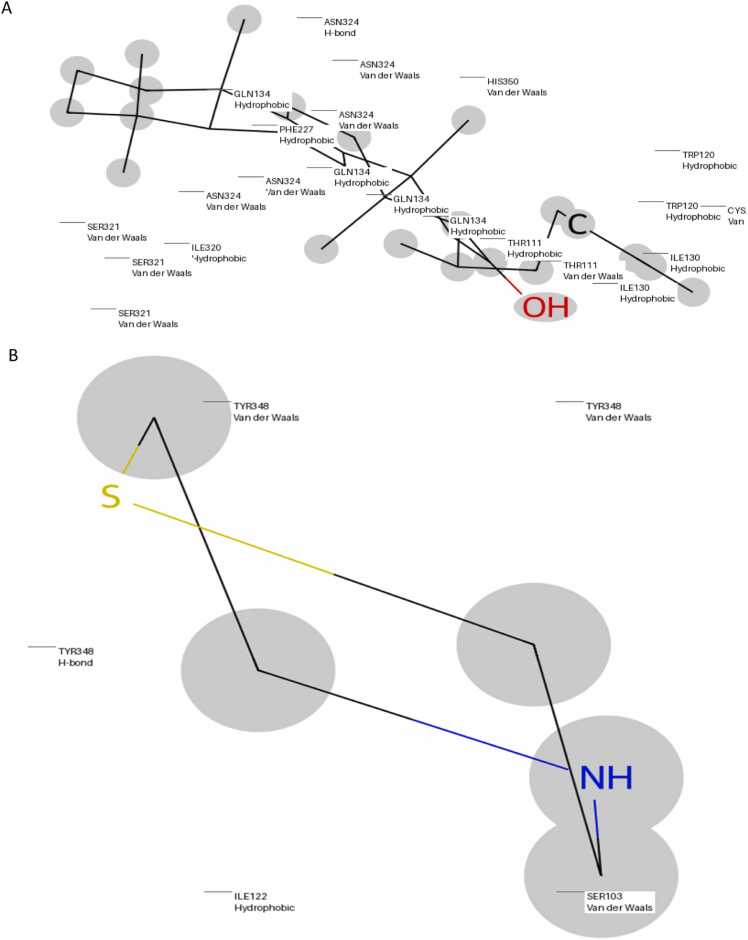
2D representation of molecular interactions of the top-performing neem compound(Neem_PDB_101602319:(5S,10S,13S,14S,17S)-4,4,10,13,14-pentamethyl-17-[(2S)-6-methylhept-5-en-2-yl]-1,2,3,5,6,7,11,12,15,17-decahydrocyclopenta[*a*]phenanthren-16-one) on 4S0V (A), and the top-performing neem compound (Neem_PDB_101257:Tirucallol) on 6V9S (B).

Orexin receptor heatmap analysis (Fig. 9, Fig. 10) depicts the relative interaction tendencies of neem-derived compounds with orexin receptor subtypes 6V9S and 4SOV. While Neem_PDB_163184214 (Meliatetraolenone) only targets ASN318 in 6V9S, compound Neem_PDB_54580354 selectively targets GLN134 in 4SOV (number of interactions >90). These differential interaction patterns confirm the subtype selectivity potential of the chemical library. Structural examination of the complexes between the receptor and ligand (Fig. 11a-b) reveals the molecular origin of this selectivity. While TYR348 is a two-fold functional anchor, the hydrophobic network of interactions in Fig. 11a, stabilized by GLN134 and reinforced by an OH-mediated hydrogen bond, differs from the S/NH-centered binding mode in Fig. 11b. Cross-referenced against interaction heatmaps, these ancillary binding processes indicate that selective regulation can be enabled by strategic functionalization of the neem scaffold [20], [22]. The high-frequency involvement of TRP120, PHE227, and HIS350 in diverse chemicals indicates evolutionary conservation of binding hotspots amenable to being targeted for the rational design of drugs. The findings oppose the traditional "one-pharmacophore" philosophy towards orexinergic modulators and propose subtype selectivity to arise through moderate variation in interaction networks, rather than radical alterations in structures [23], [25]. This redirection of emphasis allows for the possibility of synching up optimal treatments with ideal sleep-wake regulation and low off-target activity. The found key binding residues (TYR348, GLN134, TRP120) coordinate with evolutionarily conserved hotspots from crystallography analyses to confirm the translational relevance of our predictions for interactions. Our finding of orexinergic receptor modulation completes this well-crafted pharmacological profile, further advancing neem's status as a multifunctional phytochemical treasure chest for precision medicine applications to neurological, metabolic, and systemic therapeutic interventions [39], [40].

**Table:** Neem compounds identification against their identifiers for Fig. 9.

**Identifier**	**Compound Name**
Neem_PDB_101602319	(5S,10S,13S,14S,17S)−4,4,10,13,14-pentamethyl−17-[(2S)−6-methylhept−5-en−2-yl]−1,2,3,5,6,7,11,12,15,17-decahydrocyclopenta[*a*]phenanthren−16-one
Neem_PDB_101257	Tirucallol
Neem_PDB_54580354	7-Benzoylnimbocinol
Neem_PDB_102275331	[(1 R,4bR,5S,6 R,8 R,10S,10aR,10bR,12aR)−6,8-diacetyloxy−1-(furan−3-yl)−5-hydroxy−4b,7,7,10a,12a-pentamethyl−3-oxo−1,5,6,6a,8,9,10,10b,11,12-decahydronaphtho[2,1-*f*]isochromen−10-yl] (*E*)−3-phenylprop−2-enoate
Neem_PDB_6443005	Nimbolin B
Neem_PDB_12004512	Gedunin
Neem_PDB_5280794	Stigmasterol
Neem_PDB_94204	24-Methylenecycloartanol
Neem_PDB_76316558	Isomeldenin
Neem_PDB_14492795	Nimbaflavone

**Table:** Neem compounds identification for Fig. 10.

**No.**	**Compound ID**	**Chemical Name**
1.	Neem_PDB_101257	Tirucallol
2.	Neem_PDB_12072821	[(5 R,7 R,8 R,9 R,10 R,13S,17 R)−17-(2-methoxy−5-oxo-2H-furan−4-yl)−4,4,8,10,13-pentamethyl−3-oxo−5,6,7,9,11,12,16,17-octahydrocyclopenta[*a*]phenanthren−7-yl] acetate
3.	Neem_PDB_12004512	Gedunin
4.	Neem_PDB_184937	Nimolicinol
5.	Neem_PDB_54580354	7-Benzoylnimbocinol
6.	Neem_PDB_76316558	Isomeldenin
7.	Neem_PDB_163184214	Meliatetraolenone
8.	Neem_PDB_94204	24-Methylenecycloartanol
9.	Neem_PDB_169088	Nimbidin
10.	Neem_PDB_184310	Isonimocinolide

## 4 Conclusion

This detailed consideration of neem compounds as likely orexinergic receptor modulators has identified rather a significant number of findings of importance. Our molecular property screening guaranteed that many of the investigated compounds possess respectable drug-like attributes, meeting Lipinski's rule of five through appropriate molecular masses (350–450 Da) and lipophilicity (LogP 0–2) to qualify for pharmaceutical use. Differential binding energy curves between receptor subtypes 4S0V and 6V9S indicate neem compounds exhibit selective binding to the 6V9S receptor, on which basis subtype-selective modulator design could be based. Hierarchical cluster analysis also indicated divergent clustering patterns of neem compounds, with individual members having patterns of receptor selectivity that may be an objective for drug design. Identification of lead compounds such as Neem_PDB_10257 (Tirucallol) and Neem_PDB_12072821 [(5 R,7 R,8 R,9 R,10 R,13S,17 R)-17-(2-methoxy-5-oxo-2H-furan-4-yl) −4,4,8,10,13-pentamethyl-3-oxo-5,6,7,9,11,12,16,17-octahydrocyclopenta[*a*]phenanthren-7-yl] acetate) with dramatic differences in binding energy between receptor subtypes are good leads to selectively modulate the orexinergic system. Interaction analysis indicated that selectivity is due to moderate differences in interaction networks rather than radical structure changes, denying the traditional "one-pharmacophore" philosophy of orexinergic modulators. Conserved binding hotspots such as TYR348, TRP120, PHE227, and HIS350 are rational drug design targets. These findings collectively suggest that molecules derived from neem are a treasure house of new scaffolds towards the identification of selective orexinergic receptor modulators with improved therapeutic profiles. By selectively modulating particular receptor subtypes, such molecules are poised to circumvent the limitations of conceiving targeted therapy for sleep-wake disorders with fewer off-target liabilities. Future research will need to focus on experimental validation of these computer predictions and structural refinements of the lead compounds thus identified to enhance their selectivity, efficacy, and pharmacokinetics.

## CRediT authorship contribution statement

**Adewale Adetola I:** Visualization, Investigation. **Osunsanmi Foluso O:** Supervision. **Idowu Olajumoke T.:** Visualization, Investigation. **Oyinloye Babatunji E:** Supervision. **Samuel S. Agboola:** Supervision. **Basiru O. Ajiboye:** Supervision. **Agboola Oluwaseun E:** Writing – review & editing, Writing – original draft, Software, Methodology, Formal analysis, Data curation, Conceptualization. **Ayinla Zainab A:** Validation, Software, Methodology, Conceptualization. **Agboinghale Precious E:** Supervision. **Oluranti E. Olaiya:** Visualization, Investigation. **Oyebamiji Abel K:** Supervision. **Oyinloye Oluwatoyin M:** Visualization, Investigation. **Fajana Omotola M:** Visualization, Investigation.

## Ethics approval and consent to participate

Not Applicable

## Consent for publication

Not Applicable.

## Funding

Not Applicable

## Declaration of Competing Interest

The authors declare that they have no known competing financial interests or personal relationships that could have appeared to influence the work reported in this paper.

## Data Availability

Data will be made available on request.
